# Non-inferiority of automated deep learning-based [^18^F]FDG PET/CT tumour volume compared to manual GTV for prognostic modelling in head and neck cancer

**DOI:** 10.1186/s13550-026-01377-0

**Published:** 2026-02-06

**Authors:** David G. Kovacs, Katrin Håkansson, Jacob Rasmussen, Barbara M. Fischer, Flemming L. Andersen, Claes N. Ladefoged

**Affiliations:** 1 Department of Clinical Physiology and Nuclear Medicine, Rigshospitalet, Copenhagen, Denmark; 2 Department of Clinical Medicine, Faculty of Health and Medical Sciences, University of Copenhagen, Copenhagen, Denmark; 3 Department of Oncology, Centre of Cancer and Organ Diseases, Copenhagen University Hospital Rigshospitalet, Blegdamsvej 9, 2100 Copenhagen, Denmark; 4 Department of Otorhinolaryngology, Head & Neck Surgery and Audiology, Copenhagen University Hospital - Rigshospitalet, Blegdamsvej 9, 2100 Copenhagen, Denmark; 5 Department of Applied Mathematics and Computer Science, Technical University of Denmark, Lyngby, Denmark

**Keywords:** Head and neck cancer, [^18^F]FDG PET/CT, Deep learning, Tumour volume delineation, Risk stratification

## Abstract

**Background:**

Manual segmentation of gross tumour volumes (GTV) on [^18^F]FDG PET/CT is time-consuming and subject to interobserver variability, limiting its scalability for prognostic modelling in head and neck cancer. We investigated whether deep learning-based PET tumour volumes (AI-PET-GTV) could replace manually defined GTVs in risk prediction models for loco-regional failure (LRF) and distant metastasis (DM).

**Results:**

Using competing risk regression, we tested whether AI-PET-GTV was non-inferior to manual GTV in predicting LRF, with the primary outcome being area under the receiver operating characteristic curve (AUC) at 3 years, using a non-inferiority margin of 5 percentage points. AI-PET-GTV achieved a 3-year AUC of 72.9% (95% CI: 67.9–77.9%) compared to 72.8% (95% CI: 67.8–77.9%) for manual GTV (p = 0.02). At 1 year, AUCs were 77.3% (95% CI: 72.2–82.4%) and 76.9% (95% CI: 71.9–82.0%) for AI and manual GTV, respectively (p = 0.02). Similar patterns were observed for DM prediction at 1 and 3 years (all p < 0.01), and Brier scores also favoured AI-PET-GTV at both timepoints (p < 0.02). Stratification based on predicted risk yielded nearly identical cumulative incidence estimates. For example, the 3-year cumulative incidence of LRF in the high-risk group was 38.4% (95% CI: 32.6–44.2%) for both models.

**Conclusions:**

Automated deep learning-based PET tumour volumes are non-inferior to manual GTVs for prognostic modelling of LRF and DM in head and neck cancer. These findings support clinical implementation of AI-derived volumes for reproducible, scalable, and earlier risk stratification in oncology workflows.

**Supplementary Information:**

The online version contains supplementary material available at 10.1186/s13550-026-01377-0.

## Background

2-[^18^F]fluoro-2-deoxy-D-glucose positron emission tomography/computed tomography ([^18^F]FDG-PET/CT) plays a central role in head and neck cancer (HNC) management, informing staging, radiotherapy planning, and treatment response assessment [1, 2]. Beyond its role in detecting primary tumours, nodal spread, and distant metastases, PET/CT is increasingly used to guide radiotherapy, with PET-defined gross tumour volumes (PET-GTV) serving as a foundation for manual gross tumour volume (GTV) delineation in some centres [3, 4].

Manual GTV is an established prognostic biomarker for loco-regional failure (LRF) and distant metastases (DM) [5], and risk stratification based on tumour volume may inform treatment intensification, de-escalation, or patient selection for emerging therapies [6]. Accurate early identification of high-risk patients is therefore clinically valuable across a range of decision points.

We recently developed a deep learning model for automated PET-GTV segmentation (AI-PET-GTV), trained on a curated single-centre cohort of 835 patients with HNC [7, 8]. The model closely approximates expert-defined volumes and supports robust biomarker extraction and risk prediction.

In the Capital Region of Denmark, [^18^F]FDG-PET/CT scans are acquired in treatment position and used in routine radiotherapy planning. Manual GTV and clinical target volumes (CTV) are defined via multidisciplinary review. However, manual delineation introduces subjectivity and delays risk stratification. AI-PET-GTV may enable earlier, standardised prognostic assessment while reducing interobserver variability.

In this study, we evaluate whether AI-PET-GTV can replace manually defined GTV in a validated prognostic model for LRF and DM [5]. Using a non-inferiority design, we compare predictive performance of deep learning-based and manual volumes in a retrospective cohort of patients treated with curative-intent chemoradiotherapy. Our goal is to assess the clinical utility of AI-PET-GTV for risk stratification and to support its integration into future prognostic workflows.

## Methods

AI-PET-GTV delineation model used in this study is approved for tumour volume decision support and biomarker extraction at Rigshospitalet by the Department of Digital Regulation, Center for Medical Technology, Capital Region of Denmark (journal no. 24056754) [9].

### Study design and patient population

This retrospective study included patients with HNC referred for [^18^F]FDG-PET/CT-guided radiotherapy at Rigshospitalet, Department of Oncology, Section for Radiotherapy, between 2005 and 2012. Eligible patients received radiotherapy as primary treatment, supplemented with cisplatin when feasible. All patients were 18 years of age or older. Further patient and disease characteristics have been described previously [7].

The study builds on two previously published models:

- A prognostic model developed by Håkansson et al. [5], which was fitted on a cohort of 540 patients scanned between 2005 and 2012 (used here as the evaluation cohort).
- A deep learning-based AI-PET-GTV segmentation model developed under registration ISRCTN16907234 [7], trained on an independent single-centre cohort of 835 HNC patients scanned between 2014 and 2019 (training cohort).

In this study, the AI-PET-GTV model was applied to the evaluation cohort to assess whether deep learning-derived tumour volumes could replace manually defined GTV in the validated risk model. Treatment and follow-up details for this cohort are described in Rasmussen et al. [10]. The statistical analysis plan mirrored that of Håkansson et al. and was defined prior to data access.

### Imaging

All patients in the evaluation cohort underwent radiotherapy planning scans on older-generation integrated PET/CT systems: Biograph 40 TruePoint 40 HD-PET and Biograph 16 TruePoint (Siemens Healthineers, Erlangen, Germany), and Discovery LS, 4 Slice (GE Healthcare, Chicago, IL, USA). Scans were acquired between 2005 and 2012. Patients were immobilised in treatment position using moulded 5-point masks, and the CT component of the PET/CT scan was used for treatment planning. The first 35 cm from the apex of the head were included as input to the AI-PET-GTV delineation.

PET images were reconstructed using ordered-subsets expectation maximisation (OSEM). Point-spread function (PSF) correction was introduced clinically in 2010 and applied accordingly. Further acquisition details are provided in Håkansson et al. [5] (evaluation cohort) and Kovacs et al. [7] (training cohort used for model development).

### AI-PET-GTV and manual GTV delineation

In this study, all PET-based tumour volume delineations were generated using the AI-PET-GTV segmentation model developed by Kovacs et al. [7]. The following describes the procedure used to generate the manual PET-GTVs that served as training data for the model, providing insight into the type of tumour volume the algorithm was trained to replicate.

Manual PET-GTVs were delineated directly on the PET image, optionally with reference to the corresponding CT image. Iso-contours were visually adapted to follow the steepest gradient between FDG-avid malignant regions and surrounding tissue, excluding areas of physiological or non-malignant uptake. No fixed SUV threshold was applied during this process [7].

In clinical routine, these manual PET-GTVs were delineated by nuclear medicine physicians and subsequently reviewed by radiation oncologists and radiologists. The manual PET-GTV served as a guide for manual gross tumour volume (GTV) delineation, which was performed according to national guidelines defined by the Danish Head and Neck Cancer Group (DAHANCA) [11]. Importantly, the manual PET-GTVs originally created for the manual GTV delineations used this study were created without access to the AI-generated segmentations.

### Prediction of loco-regional failure and distant metastases

We used a competing risk model to predict loco-regional failure (LRF) and distant metastasis (DM), treating death with no evidence of disease as a competing event. This endpoint was not analysed separately, as tumour volume was not included as a predictor of death in the original model by Håkansson et al. [5].

The model included pre-specified clinical variables (tumour subsite, T stage, N stage, smoking status, age, and performance status) together with tumour volume. Predictions based on the original, manually defined GTV were reproduced identically to those in Håkansson et al. [5]. To evaluate the deep learning based alternative, we substituted the manual GTV with AI-PET-GTV while keeping all other inputs unchanged. Tumour volume (manual GTV-based or AI-PET-GTV-based) was the only difference between the two prediction models.

### Statistics

The primary outcome was the area under the receiver operating characteristic curve (AUC) at three years for predicting LRF [12]. Secondary outcomes included AUC at one and five years for LRF and DM, as well as Brier scores [13] for all three time points and both endpoints.

To evaluate whether AI-PET-GTV-based predictions were non-inferior to manual GTV-based predictions, we conducted non-inferiority tests on these performance metrics. The null hypothesis stated that AI-PET-GTV was inferior to manual GTV, with AUC or Brier score differences exceeding predefined non-inferiority margins. The alternative hypothesis stated that AI-PET-GTV was non-inferior, with performance within those margins. The non-inferiority margins were pre-specified as an absolute AUC difference of 0.05 and an absolute difference of 0.02 for the Brier score. These thresholds were derived empirically from the uncertainty observed in the reference analysis by Håkansson et al. [5], which used the same patient cohort and modelling framework. Because the present study evaluates a substitute predictor within the same fixed dataset, substantially smaller margins would fall below the practical resolution of the study and would require a larger cohort to test with adequate precision. From a clinical and operational perspective, we considered an absolute AUC difference of 0.05 to represent a small difference in discrimination, and therefore an acceptable potential loss in performance in exchange for replacing manual GTV delineation with a fully automated tumour volume measure.. Our analysis replicated that setup, replacing manual GTV with AI-PET-GTV as the only modified input, with causes of patient exclusion limited to data corruption (missing scans, incomplete transfers and technical issues during format conversion). One-tailed p-values were used for non-inferiority testing. Here, a one-tailed p-value < 0.05 provides evidence of non-inferiority relative to the pre-specified margins (i.e., that performance is not worse than GTV by more than 0.05 in AUC or 0.02 in Brier score), rather than evidence of superiority.Practically, this tests whether replacing manual GTV with automated AI-PET-GTV yields prognostic models with discrimination and overall accuracy that are not meaningfully worse for separating patients into different predicted risk levels. Testing was conducted using a custom R script (version 4.4.2), provided in the Supplementary Material.

To characterise the relationship between manual GTV and AI-PET-GTV volumes, we computed Spearman correlation coefficients and performed paired t-tests. Given the conceptual differences between the delineation methods, direct equivalence was not expected.

To assess model-based stratification, we computed Aalen–Johansen estimates [14] of cumulative incidence for LRF and DM, stratifying patients into high- and low-risk groups according to the median predicted risk from each competing risk model, using the absolute risk regression framework described by Gerds et al. [15]. High- and low-risk classification was performed separately for the manual GTV-based and AI-PET-GTV-based models. All statistical tests used a significance level of 0.05.

## Results

### Patient inclusion and tumour detection

**Fig. 1:**
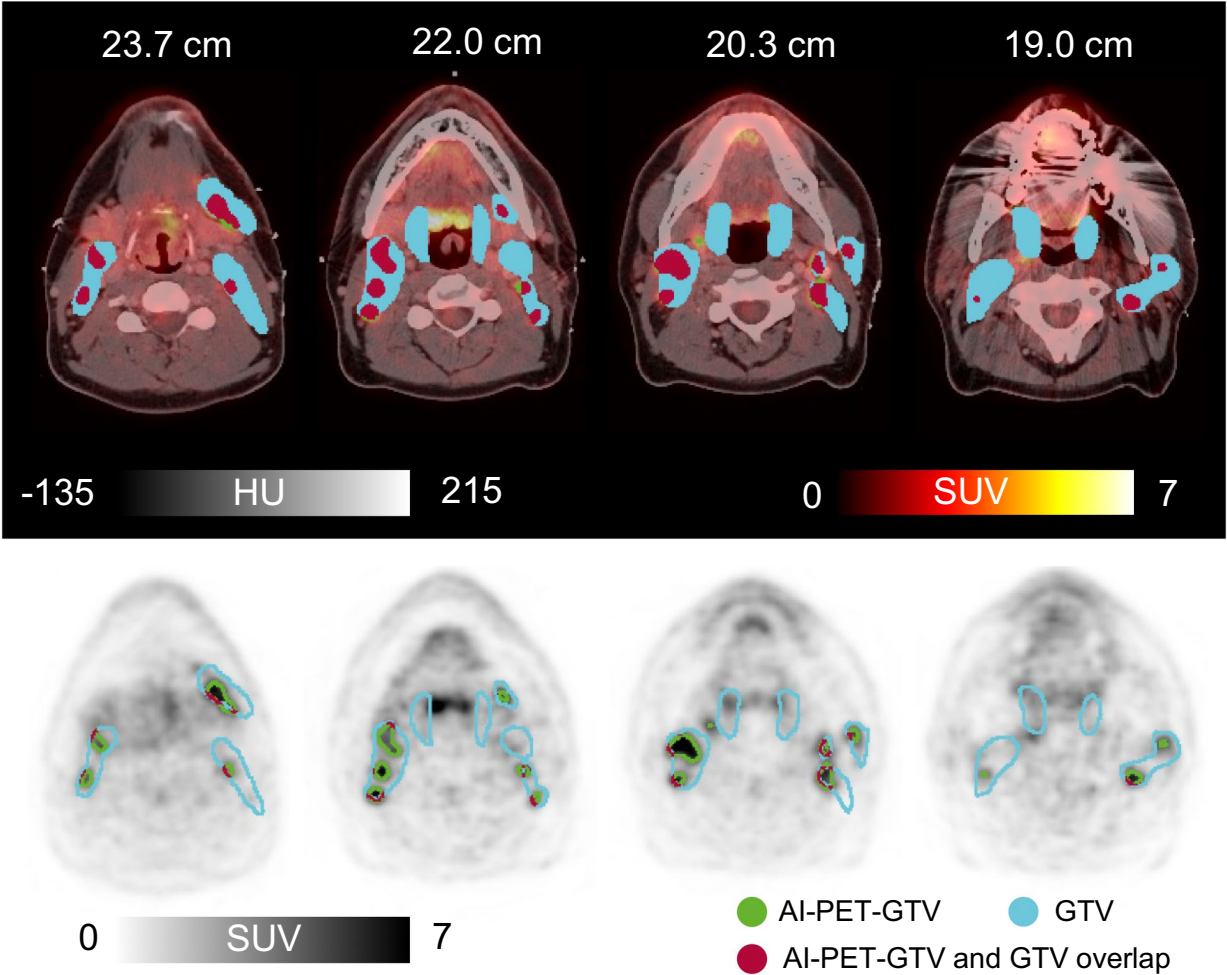
Clinical PET-CT scan from 2005 of a 40-year-old man with p16-positive oropharyngeal cancer. The patient was an active smoker at the time of imaging and did not receive cisplatin-based treatment. The axial images show the deep learning-based AI-PET-GTV delineation (green): the top panel displays fused PET-CT images (Hounsfield units, HU), while the bottom panel presents PET images alone (standardized uptake value, SUV). The manual GTV is shown in blue, and the overlapping region between manual GTV and AI-PET-GTV is shown in red. AI-PET-GTV volume was 29.0 cm^3^ compared to a manually delineated GTV of 141.2 cm^3^. The patient developed distant metastases 516 days after completion of treatment. This case was acquired independently in 2005 using different hardware, protocols, and annotations than those used for model development. The value shown above each slice represents the cranio-caudal distance (in cm) from the apex of the head. AI = artificial intelligence

We included a total of 560 patients in our analysis. Twenty patients were not included due to data corruption (e.g. missing scans and incomplete transfers (n = 19), or technical issues during format conversion (n = 1)). The remaining 540 (96%) patients were used to derive AI-PET-GTV volumes for subsequent analysis. An example of AI-PET-GTV, overlaid on clinical PET/CT images, is shown in Fig. 1 to illustrate the type of output used in the prognostic model. Among the 540 patients included in the analysis, 145 experienced LRF, 57 experienced DM, and 87 experienced deaths with no evidence of disease. Additionally, 251 patients were right-censored. Combined T, N and M failure is reported in [5].

### Comparison of risk models using standard and automated biomarkers

The comparison between the risk models including manual GTV or AI-PET-GTV reveals that the same predictors were significant across both models (Table 1), with the tumour volume itself being a significant predictor in both models. For LRF, significant predictors included being a current smoker, tumour subsite (Cavum oris, Hypopharynx, Larynx, and Oropharynx, p16-negative), and tumour volume. For DM, significant predictors were tumour volume and having a non-p16-positive oropharynx tumour. Predictors that were significant in the manual GTV model remained significant in the AI-PET-GTV model, with only slight differences in hazard ratios compared to Håkansson et al., likely due to the exclusion of 20 patients because of data corruption. For LRF, the hazard ratio (HR) for tumour volume is slightly higher in the manual GTV model (HR = 1.57, 95% CI: 1.28–1.91, p < 0.001) compared to the AI-PET-GTV model (HR = 1.43, 95% CI: 1.19–1.71, p < 0.001).

**Table 1:** Comparison of cause-specific risk models including manual GTV and AI-PET-GTV: hazard ratios and predictor significance

Predictor	Unit	Manual GTV model (original)		AI-PET-GTV model (new)	
		Hazard ratio [95% CI]	P-value	Hazard ratio [95% CI]	P-value
Loco-regional failure					
Tobacco	Reference: Never/previous smoker	1.00 (reference)		1.00 (reference)	
	Current smoker	1.49 [1.04–2.16]	0.03	1.50 [1.04–2.16]	0.03
Tumour subsite	Reference: Oropharynx, p16-positive	1.00 (reference)		1.00 (reference)	
	Cavum oris	3.90 [2.07–7.35]	<0.001	3.46 [1.83–6.52]	<0.001
	Hypopharynx	5.01 [2.86–8.78]	<0.001	4.40 [2.52–7.68]	<0.001
	Larynx	2.66 [1.47–4.80]	<0.01	2.42 [1.35–4.33]	<0.01
	Oropharynx, p16-negative	2.54 [1.42–4.55]	<0.01	2.48 [1.38–4.43]	<0.01
T-stage	T-stage	1.10 [0.89–1.35]	0.39	1.08 [0.87–1.33]	0.49
N-stage	Reference: N0	1.00 (reference)		1.00 (reference)	
	N1-N2b	1.01 [0.55–1.84]	0.98	1.04 [0.57–1.91]	0.89
	N2-N3 (incl. N2 for p16+OPSCC)	1.35 [0.73–2.49]	0.34	1.40 [0.76–2.60]	0.28
Tumour volume	log (cm3)	1.57 [1.28–1.91]	<0.001	1.43 [1.19–1.71]	<0.001
Distant metastasis					
Tobacco	Reference: Never/previous smoker	1.00 (reference)		1.00 (reference)	
	Current smoker	1.20 [0.69–2.09]	0.53	1.16 [0.66–2.04]	0.59
Tumour subsite	Reference: Oropharynx, p16 positive	1.00 (reference)		1.00 (reference)	
	Oropharynx, p16-negative/other tumour	2.03 [1.06–3.87]	0.03	1.93 [1.01–3.67]	0.046
T-stage	T-stage	1.05 [0.76–1.45]	0.77	0.92 [0.66–1.29]	0.64
N-stage	Reference: N0	1.00 (reference)		1.00 (reference)	
	N1-N2b	0.76 [0.27–2.13]	0.60	0.65 [0.23–1.80]	0.41
	N2-N3 (incl. N2 for p16+OPSCC)	0.90 [0.31–2.58]	0.84	0.76 [0.28–2.11]	0.60
Tumour volume	log (cm3)	2.35 [1.68–3.28]	<0.001	2.62 [1.90–3.62]	<0.001

Significant predictors (p < 0.05) are highlighted in bold. All models were stratified and adjusted using the pre-defined covariates specified by Håkansson et al. [5]. These covariates were originally selected by clinician consensus. The results demonstrate that the same predictors are significant across both models, with no predictors being significant in one model but not the other. This consistency supports the potential for AI-PET-GTV to replace manual GTV as a predictor

GTV, gross tumour volume; AI-PET-GTV, automated PET-based GTV derived from the biomarker model; T-stage, tumour stage (UICC staging guidelines); N-stage, nodal stage (UICC staging guidelines); OPSCC, oropharyngeal squamous cell carcinoma; N/A, not applicable

For DM, tumour volume has the largest effect size of all predictors in both models (manual GTV: HR = 2.35, 95% CI: 1.68–3.28, p < 0.001; AI-PET-GTV: HR = 2.62, 95% CI: 1.90–3.62, p < 0.001) and having a non-p16-positive oropharynx tumour also showed a significant effect size (manual GTV: HR = 2.05, 95% CI: 1.07–3.91, p = 0.03; AI-PET-GTV: HR = 2.07, 95% CI: 1.07–4.01, p = 0.03), while being a smoker was not significant in either model.

### Comparative predictive performance of the models

**Fig. 2:**
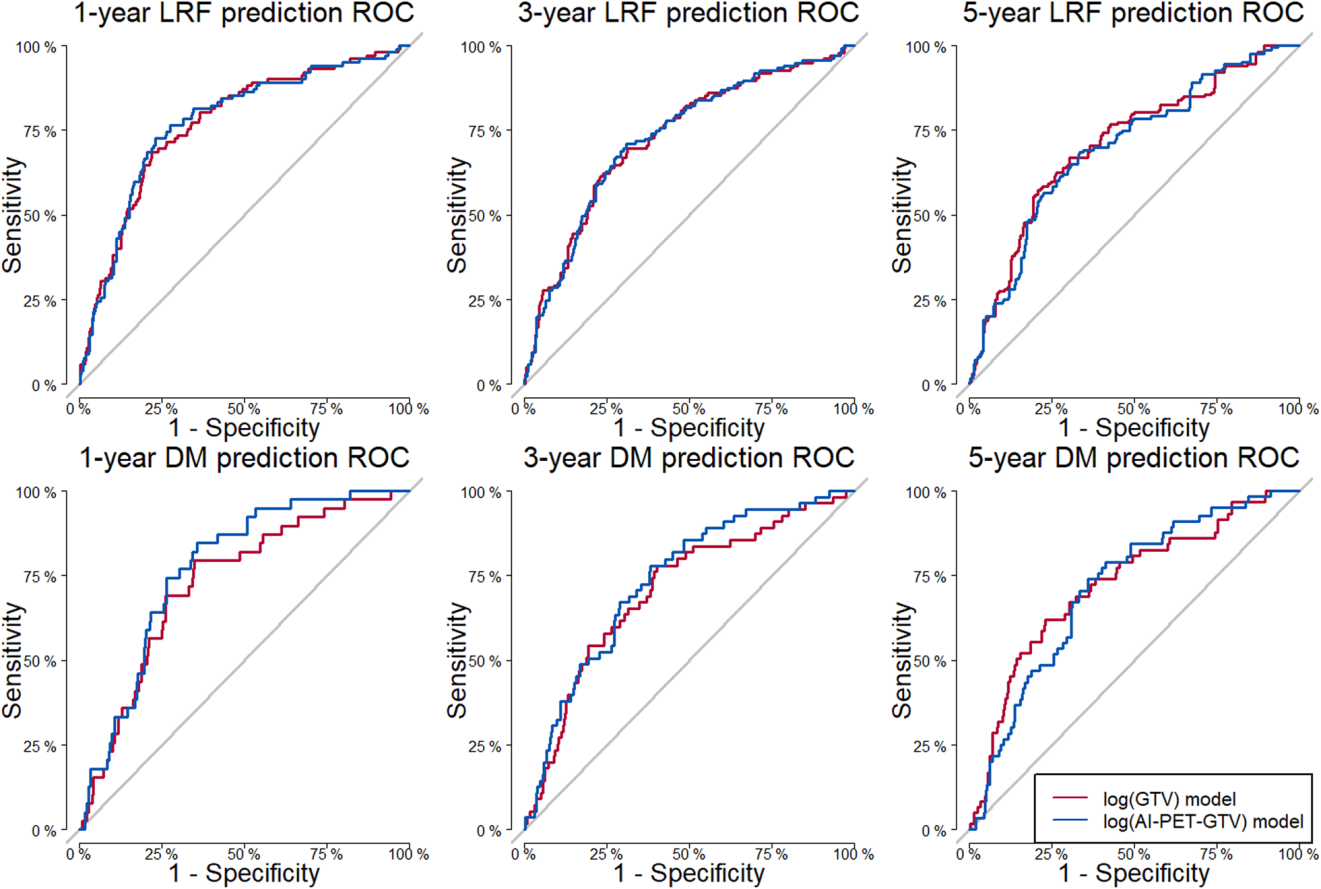
Receiver operating characteristic (ROC) curves comparing the predictive performance of two prognostic models: one using manual gross tumour volume (GTV, red) and the other using deep learning-derived PET-GTV (AI-PET-GTV, blue) for predicting loco-regional failure (LRF) and distant metastasis (DM) at 1, 3, and 5 years. At 1 and 3 years, non-inferiority of the AI-PET-GTV model was confirmed (all p < 0.05), supporting its comparability to the manual GTV-based model. The similar curve shapes across time points visually support the finding that deep learning-based delineations provide predictive performance equivalent to manual GTV

The primary outcome of the study was the area under the curve (AUC) for loco-regional failure (LRF) at 3 years (Table 2, Fig. 2). At this time point, the AI-PET-GTV model demonstrated an AUC of 72.9%, identical to the manual GTV model (72.8%), and non-inferiority was confirmed at the predefined margin of 5 percentage points (p = 0.02). This establishes that AI-PET-GTV performs comparably to manual GTV for 3-year LRF prediction.

**Table 2:** Comparative predictive performance of the manual GTV and AI-PET-GTV models for loco-regional failure (LRF) and distant metastasis (DM) at 1, 3, and 5 years

Time (years)	Manual GTV model (original)	AI-PET-GTV model (new)	
	value [95% CI]	value [95% CI]	P-value for non-inferiority
LRF - AUC			
1	77.0 [71.9–82.0]	77.3 [72.2–82.4]	0.02
3	72.8 [67.8–77.9]	72.9 [67.9–77.9]	*0.02
5	70.9 [62.0–79.8]	69.9 [60.7–79.1]	0.19
LRF – Brier Score			
1	0.13 [0.11–0.15]	0.13 [0.11–0.15]	0.02
3	0.17 [0.15–0.18]	0.17 [0.15–0.18]	<0.01
5	0.19 [0.16–0.22]	0.19 [0.16–0.22]	0.09
DM - AUC			
1	73.0 [67.8–78.1]	77.1 [71.8–82.4]	<0.001
3	70.8 [65.2–76.4]	73.6 [67.8–79.3]	<0.001
5	72.7 [62.0–83.4]	72.2 [58.2–86.2]	0.20
DM – Brier Score			
1	0.07 [0.05–0.08]	0.07 [0.05–0.08]	<0.01
3	0.09 [0.07–0.11]	0.09 [0.07–0.11]	0.02
5	0.09 [0.07–0.11]	0.10 [0.08–0.11]	0.16

Metrics include area under the curve (AUC) and Brier score, each with 95% confidence intervals (CI). Reported p-values are from one-sided non-inferiority tests, evaluating whether the AI-PET-GTV model performs no worse than the manual GTV model beyond predefined margins (5 percentage points for AUC, 0.02 for Brier score). A p-value < 0.05 indicates non-inferiority. The primary endpoint (3-year LRF AUC) is marked with an asterisk

GTV, gross tumour volume; AI-PET-GTV, automated PET-based GTV derived from the biomarker model; LRF, loco-regional failure; DM, distant metastasis; AUC, area under the curve; CI, confidence interval

Non-inferiority was also demonstrated at 1 year for both AUC and Brier score (AUC: 77.3% vs. 77.0%, p = 0.02; Brier score: 0.13 vs. 0.13, p = 0.02). At 5 years, the AUC and Brier scores remained numerically similar between models, but the confidence intervals were wider, and non-inferiority could not be confirmed (AUC p = 0.19; Brier score p = 0.09).

For distant metastases (DM), the AI-PET-GTV model showed numerically higher AUCs than the manual GTV model at both 1 and 3 years (1 year: 77.1% vs. 73.0%; 3 years: 73.6% vs. 70.8%), and non-inferiority was statistically confirmed at both time points (p < 0.001). At 5 years, the AUCs remained comparable (72.2% vs. 72.7%), but non-inferiority was not confirmed (p = 0.20), likely due to limited sample size. Brier scores for DM were closely aligned across all time points, with non-inferiority demonstrated at 1 and 3 years.

Together, these results confirm that AI-PET-GTV-based predictions are non-inferior to manual GTV-based predictions at the primary 3-year endpoint, and at the 1-year mark, across both outcomes and performance metrics. Calibration plots for 3-year cause-specific predicted risk of LRF is provided in the Supplementary Material (Supplementary Fig. S1) for both the manual GTV and AI-PET-GTV models.

### Tumour volume association

**Fig. 3:**
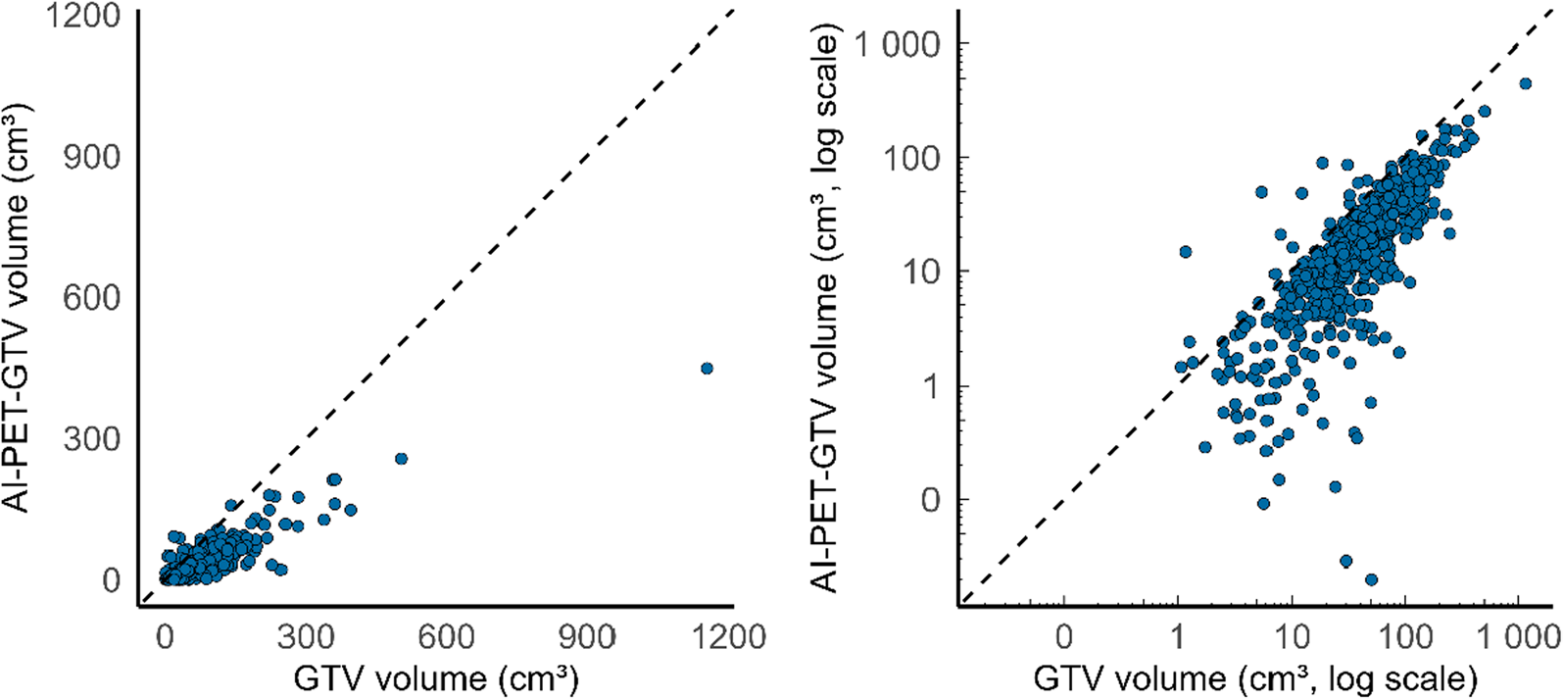
Scatter plots comparing manual gross tumour volumes (GTV) between manual expert contours and deep learning-based PET-GTV predictions (AI-PET-GTV). Each point represents a patient scan. The left plot shows volumes on a linear scale; the right plot shows volumes on a log–log scale. The dashed identity line (y = x) indicates where manual and deep learning-based volumes would be equal. A strong positive correlation was observed (Spearman ρ = 0.822, p < 2.2 × 10^–16^), although manual GTV volumes were typically larger than AI-PET-GTV volumes

Spearman’s correlation analysis (Fig. 3) showed a strong positive correlation between manual GTV and AI-PET-GTV volumes (ρ = 0.822, p < 2.2 × 10⁻^1^⁶), indicating that larger manual GTVs generally corresponded to larger AI-PET-GTVs. However, as expected, systematic differences were observed, since manual GTV typically includes both FDG-avid and non-FDG-avid tumour regions (demonstrated in Fig. 1), whereas AI-PET-GTV is restricted to the PET-positive volume. A paired t-test confirmed that manual GTV volumes were significantly larger than AI-PET-GTV volumes, with a mean difference of 31.7 cm^3^ (95% CI: 27.8–35.6, p < 2.2 × 10⁻^1^⁶).

### Risk stratification agreement and differences between models

**Fig. 4:**
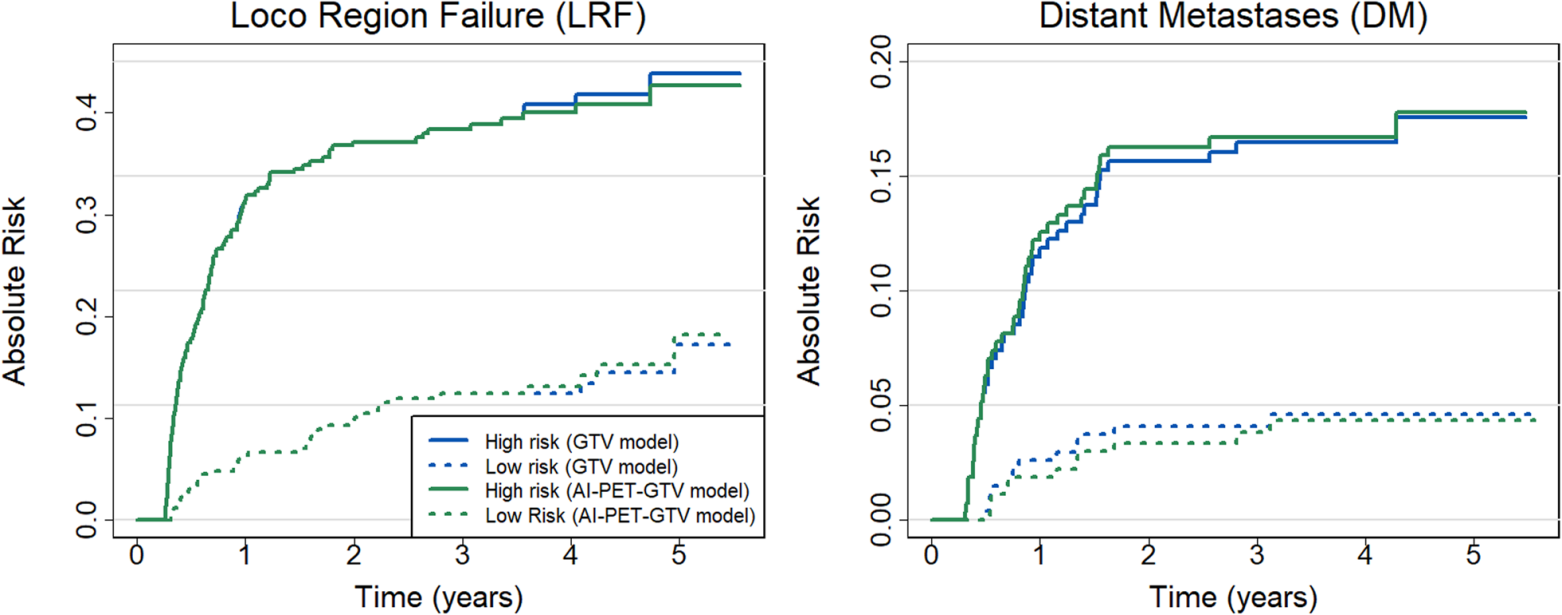
Cause-specific cumulative incidence functions (CIFs) for locoregional failure (LRF, left panel) and distant metastases (DM, right panel), stratified by predicted median 3-year risk from the competing risk models, based on 540 patients. Curves are shown for high- and low-risk groups from the manual GTV-based model (blue) and the AI-PET-GTV-based model (green). Solid lines represent high-risk groups, and dashed lines represent low-risk groups. The results illustrate the probability of LRF and DM over time, demonstrating that the AI-PET-GTV model provides risk stratification comparable to that of the manual GTV model

The cause-specific cumulative incidence functions for LRF and DM were estimated using the Aalen-Johansen method and stratified by predicted risk (Fig. 4). Patients were classified as high or low risk based on the median 3-year risk predicted by the competing risk models using manual GTV and AI-PET-GTV, respectively. Figure 4 presents these cumulative incidence curves over time, enabling further evaluation of risk patterns at multiple time points. At 3 years, the cumulative incidence of LRF was identical for both models: 38.4% (95% CI: 32.6–44.2%) in the high-risk group and 12.4% (8.4–16.4%) in the low-risk group, as shown in Fig. 4. For DM, the high-risk group had a cumulative incidence of 16.7% (12.3–21.2%) in the AI-PET-GTV model and 16.5% (12.0–20.9%) in the manual GTV model, compared to 3.8% (1.5–6.1%) and 4.1% (1.7–6.5%), respectively, in the low-risk groups.

**Fig. 5:**
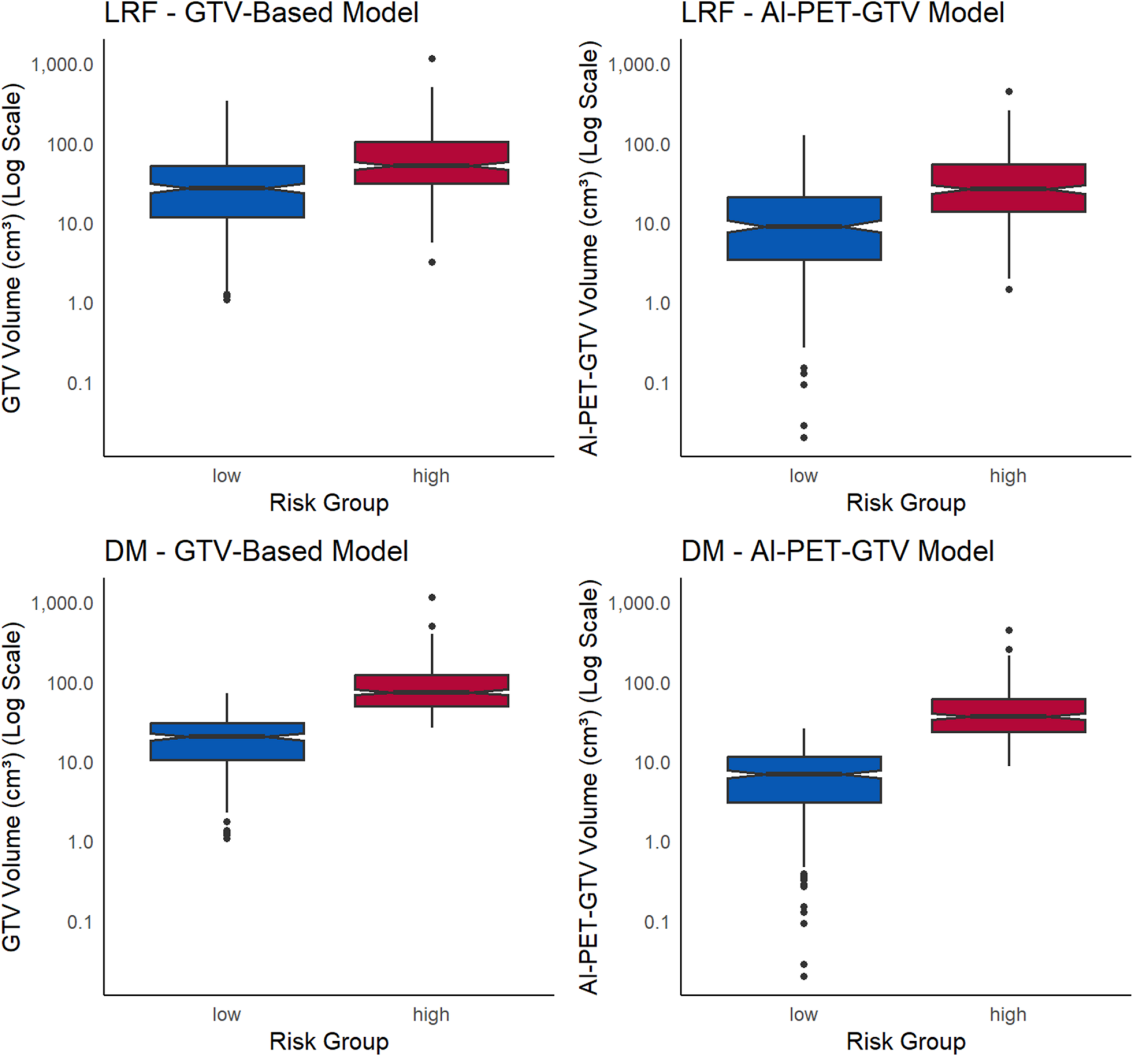
Distribution of manual GTV and AI-PET-GTV volume stratified by risk groups for loco-regional failure (LRF) and distant metastasis (DM), based on the manual GTV-based model (left) and the AI-PET-GTV model (right). Tumour volumes are shown on a logarithmic scale to improve visualisation across the full range of tumour volumes. Boxes represent the interquartile range (IQR), with the median indicated by a central line and whiskers extending to the most extreme values within 1.5 × IQR. Data points beyond the whiskers are shown individually. Notches represent approximate 95% confidence intervals around the median. The high-risk group (red) includes patients with predicted risk above the median (n = 270) for each model; the low-risk group (blue) includes those with predicted risk at or below the median (n = 270)

To support clinical interpretation, we examined tumour volumes in high- and low-risk groups, defined by stratifying patients at the median predicted risk derived from the competing risk models (Fig. 4). For locoregional failure (LRF), the tumour volumes (median [IQR]) in high- and low-risk patients were 52.2 cm^3^ (31.3–103.6) and 27.0 cm^3^ (11.9–52.1), respectively, using the manual GTV model, and 26.3 cm^3^ (13.8–54.3) and 8.9 cm^3^ (3.4–20.8) using the AI-PET-GTV model. For distant metastasis (DM), the corresponding volumes were 73.8 cm^3^ (49.3–121.4) and 20.4 cm^3^ (10.5–30.5) for the manual GTV model, and 36.3 cm^3^ (23.6–60.3) and 6.9 cm^3^ (3.1–11.4) for the AI-PET-GTV model. These values illustrate the typical tumour burden associated with high- and low-risk classifications under each model and may help clinicians interpret individual patient volumes in context. (Fig. 5).

## Discussion

This study evaluated whether automatically extracted gross tumour volumes (AI-PET-GTV) can serve as a reliable alternative to manually delineated GTV for prognostication and risk stratification in head and neck cancer. By applying an existing deep learning model to a large, independent cohort, we demonstrated that AI-PET-GTV achieved non-inferior predictive performance to manual GTV when applied in a validated multi-endpoint prognostic model [16]. At the 3-year primary endpoint, AI-PET-GTV achieved an AUC of 72.9% [67.9–77.9], showing non-inferiority compared to manual GTV (p = 0.02). Similar non-inferiority was observed at 1 year for both loco-regional failure (LRF) and distant metastases (DM), while the smaller sample size at 5 years limited statistical confirmation. Importantly, both deep learning-derived and manual volumes stratified patients into high- and low-risk groups with near-identical cumulative incidence patterns, supporting comparable clinical utility for risk stratification. We used the median predicted 3-year risk as a pragmatic threshold to form equal sized groups for comparison. In clinical use, clinically meaningful cut-offs would be selected for specific decisions and acceptable trade-offs between sensitivity and specificity.

Although AI-PET-GTV volumes were systematically smaller than manual GTV, this reflects their restriction to PET-avid regions and is consistent with known differences in delineation scope. Despite this systematic offset, manual GTV and AI-PET-GTV volumes were strongly correlated (Spearman rho 0.822, p < 0.001). Manual GTV was larger on average by 31.7 cm^3^ (95% CI: [27.8;35.6]), consistent with inclusion of non FDG-avid tumour components. Importantly, the prognostic analysis does not require volumetric equivalence, and this offset did not translate into reduced downstream performance, as reflected by non-inferior discrimination at three years and similar risk stratification patterns. An important advantage of AI-PET-GTV lies in its consistency and reproducibility: the model yields identical results for identical input, avoiding the inter-observer variability associated with manual contours [17]. Furthermore, deep learning-based predictions can be generated earlier in the clinical workflow, before manual contours are available, offering timely prognostic insights to support decision-making. Overall, our findings support AI-PET-GTV as a robust, standardised input for clinical risk modelling in head and neck cancer.

A key strength of this study is that it advances the translational validation of deep leaning in medical imaging from technical evaluation to direct assessment on patient-relevant outcomes. While earlier work focused on voxel-level agreement using the Dice coefficient as the primary endpoint [7], this study evaluated the model’s clinical utility in prognostic risk modelling for loco-regional failure and distant metastases. Validation based on clinical outcomes is essential for implementation in clinical practice, as emphasised in best-practice frameworks for trustworthy deep learning in nuclear medicine, including the RELAINCE guidelines [18] and recommendations by Saboury et al. [19]. Importantly, the evaluation was conducted on an independent cohort scanned between 2005 and 2012 using older-generation PET/CT systems, including Siemens Biograph 40 TruePoint and GE Discovery LS scanners, with non-TOF reconstruction and Gaussian post-filtering of 4–5 mm (non-PSF) or 2 mm (PSF). In contrast, the AI-PET-GTV segmentation model was developed on PET/CT scans from a separate cohort acquired on more recent Siemens Biograph mCT and Vision 600 systems using TOF, PSF correction, and a 2 mm Gaussian post-filter. This temporal and technical separation supports generalisability, reduces the risk of data leakage or performance inflation due to cohort overlap, and illustrates the model’s robustness when applied to historical data acquired under differing standards, a challenge increasingly recognised in AI validation studies [20].

In addition to demonstrating non-inferiority in predictive performance, this study highlights the potential of AI-PET-GTV to enable earlier and more consistent prognostic assessment in clinical workflows. Manual GTV delineation is typically finalised after the multidisciplinary tumour board (MTB), making manual GTV-based prognostic estimates unavailable during initial treatment discussions. In contrast, AI-PET-GTV can be derived automatically and immediately from PET/CT scans, potentially allowing risk estimates for loco-regional failure and distant metastasis to inform shared clinical decision-making at an earlier stage. These findings support the integration of AI-PET-GTV as a real-time decision support tool within risk-adapted protocols [16, 21, 22]. This is further reinforced by Dorr et al., who showed that using the OncologIQ survival prediction model during MTB discussions improved individualised decision-making in complex head and neck cancer cases [23].

While AI-PET-GTV demonstrated strong prognostic performance, clinical implementation will require addressing regulatory, technical, and operational challenges, including reproducibility, external validation, and integration into clinical workflows [24, 25]. Deep learning-based biomarkers must undergo rigorous validation, particularly when applied for a new intended use [9]. Regulatory compliance entails demonstrating reproducibility, generalisability, and clinical utility across varied populations and imaging protocols. Moreover, integration into clinical systems such as PACS and electronic health records will require standardised workflows and dedicated clinician training.

This study has limitations. Generalisability may be limited by the single-centre nature of the data, although this is partly mitigated by the use of an independent time period for validation. Generalisability to contemporary clinical settings may also be affected by differences in acquisition protocols, reconstruction methods, scanner technology, and patient selection. While our validation already spans marked technical heterogeneity, future multicentre studies should confirm performance under current protocols, and harmonization approaches can mitigate scanner and protocol related variability if needed. External validation in additional cohorts is warranted. At the 5-year time point, the sample size was insufficient to confirm non-inferiority. Although AI-PET-GTV offers a robust automated imaging biomarker, its clinical use remains exploratory and requires further evaluation. Moreover, because the confidence intervals around the AUC difference are primarily determined by the number of events observed by 3 years, a more stringent test relative to the pre-specified non-inferiority margin would require a larger dataset with more events.

We do not currently have a dedicated external validation study planned for this specific prognostic substitution analysis. However, the underlying segmentation model is publicly accessible for external testing (https://rigshospitalet-tumour-segmentation.regionh.dk/). An ideal external cohort would be independent and multicentre, include contemporary PET/CT acquisitions across different scanners and reconstruction protocols, and provide the same key clinical variables and sufficient three year follow up and event counts to support precise non-inferiority testing. Future work should assess how deep learning-derived biomarkers such as AI-PET-GTV can be integrated into real-world decision-making processes. This includes evaluating their impact on multidisciplinary discussions, treatment selection, and patient outcomes. Understanding these effects will be essential for demonstrating the clinical value of artificial intelligence deep learning tools and supporting their adoption into precision oncology workflows.

## Conclusion

This study demonstrates that AI-PET-GTV can reliably replace manual GTV in prognostic modelling, supporting its use as an automated imaging biomarker for clinical decision-making. The findings highlight its potential to enable earlier, more scalable, and consistent risk assessment in head and neck cancer workflows.

## Supplementary Information

Additional file 1.

## Abbreviations

[18F]FDG: 2-[18F]fluoro-2-deoxy-D-glucose
AI: Artificial Intelligence
AI-PET-GTV: Artificial Intelligence-derived PET-based Gross Tumour Volume
AUC: Area Under the Receiver Operating Characteristic Curve
CI: Confidence Interval
CRT: Chemoradiotherapy (include only if used in manuscript)
CT: Computed Tomography
DM: Distant Metastasis
DL: Deep Learning (include only if used in manuscript)
GTV: Gross Tumour Volume
HNC: Head and Neck Cancer
HR: Hazard Ratio
HU: Hounsfield Unit
ISRCTN: International Standard Randomised Controlled Trial Number
LRF: Loco-Regional Failure
MTB: Multi Disciplinary Board
MTV: Metabolic Tumour Volume
PACS: Picture Archiving and Communication System
PET: Positron Emission Tomography
PSF: Point Spread Function
RELAINCE: Recommendations for EvaLuation of AI for NuClear medicinE
ROC: Receiver Operating Characteristic
SUV: Standardised Uptake Value
TOF: Time of Flight
