# Supplementary material for: Non-inferiority of automated deep learning-based [18F]FDG PET/CT tumour volume compared to manual GTV for prognostic modelling in head and neck cancer
Source: EJNMMI Res. 2026 Feb 6;16:39. doi: 10.1186/s13550-026-01377-0 (PMC12972335; doi:10.1186/s13550-026-01377-0)
Supplement: Supplementary file 1 — Additional file 1. [file 13550_2026_1377_MOESM1_ESM.docx]

**Supplementary material for:**

Title: Non-inferiority of Automated Deep Learning-Based 18F-FDG PET/CT Tumour Volume Compared to Manual GTV for Prognostic Modelling in Head and Neck Cancer

Journal name: European Journal of Nuclear Medicine and Molecular Imaging

Author names:
David G. Kovacs
Katrin Håkansson
Jacob Rasmussen
Barbara M. Fischer
Flemming L. Andersen
Claes N. Ladefoged

**Corresponding author:**
David Gergely Kovacs
M.Sc in Biomedical Engineering
ORCID ID: 0000-0002-0383-1446
Address: Rigshospitalet, Copenhagen University Hospital, Blegdamsvej 9, DK-2100
Telephone, work: +45 30 47 04 33
Telephone, personal: +45 61 40 96 42
Email: [dkov0001@regionh.dk](mailto:dkov0001@regionh.dk)

**This supplementary file contains supplementary material and R code used for data analysis and figure generation in support of the findings presented in the manuscript.**

**
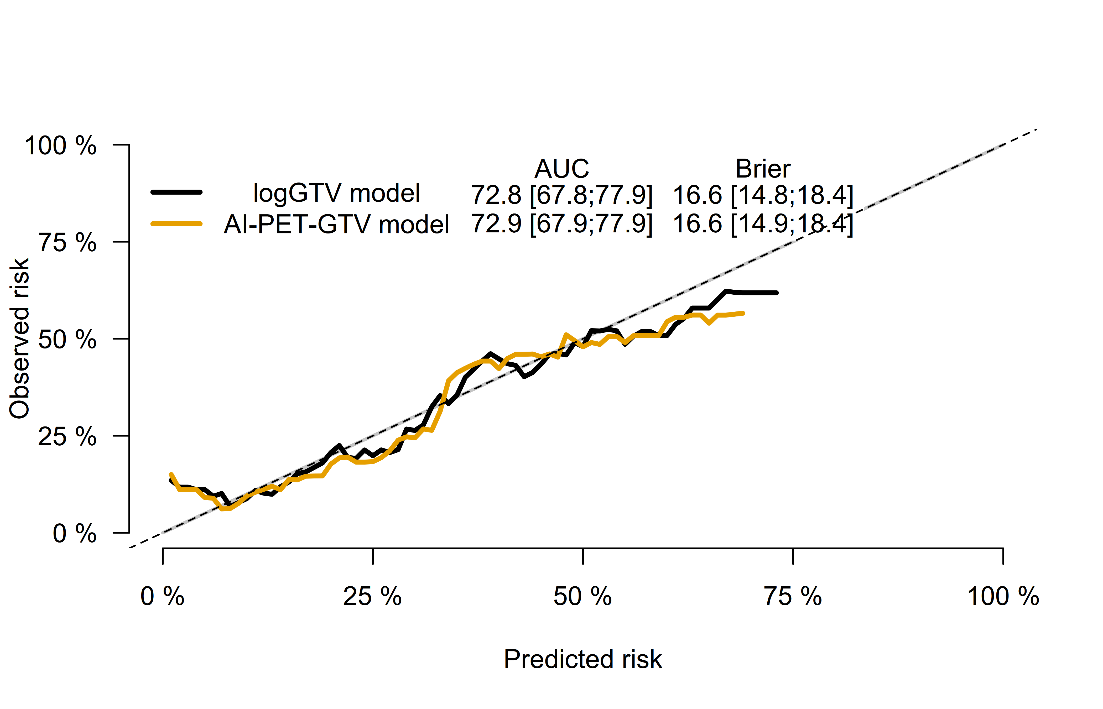
**

**Fig. S1** Calibration plot comparing predicted versus observed 3-year cumulative incidence for loco-regional failure (LRF) for the GTV and AI-PET-GTV models. The dashed diagonal indicates perfect calibration.

### 1competing_risk_models_comparison_v2

# Purpose: Fit and compare multivariable competing risk models

# for loco-regional failure (LRF) and distant metastasis (DM)

# using GTV and AI-PET-GTV biomarkers.

rm(list = ls())

# Load required libraries

library(prodlim)

library(survival)

library(riskRegression)

library(ggplot2)

library(gridExtra)

library(scales)

# -------------------------

# Script: model_comparison_analysis.R

# Purpose: Compare Cox models using logGTV, log_mtv, and PET-CT biomarkers, including n_objects_group.

# -------------------------

# Define file paths

output_analysis_folder <- "L:\\LovbeskyttetMapper01\\hnc-auto-cont-KHAA-DGK-data\\paper_2025_analysis_biomarkers\\analysis_output"

# Step 1: Load the prepared dataset

hn_final <- read.csv("L:\\LovbeskyttetMapper01\\hnc-auto-cont-KHAA-DGK-data\\merged_dataset.csv")

# due to data corruption we have to remove the study 270

hn_final <- hn_final[hn_final$StudyNo != "270", ]

hn_final$Tobacco <- relevel(factor(hn_final$Tobacco), ref = "Never/previous smoker")

hn_final$TumorSubsite <- relevel(factor(hn_final$TumorSubsite), ref = "Oropharynx, p16 positive")

hn_final$Nstage <- relevel(factor(hn_final$Nstage), ref = "N0")

hn_final$HPV <- relevel(factor(hn_final$HPV), ref = "p16+ oropharynx")

hn_final$Cisplatin <- relevel(factor(hn_final$Cisplatin), ref = "No cisplatin")

# Ensure the dataset contains required variables

required_vars <- c("time", "event", "logGTV", "log_mtv")

if (!all(required_vars %in% colnames(hn_final))) {

  stop("The dataset does not contain all required variables.")

}

# Step 2: Define Cox models

# Model with logGTV (manual GTV)

f_logGTV <- CSC(formula = list(

  Hist(time, event) ~ Tobacco + TumorSubsite + Tstage + Nstage + logGTV + strata(Cisplatin),

  Hist(time, event) ~ Tobacco + HPV + Tstage + Nstage + logGTV + strata(Cisplatin),

  Hist(time, event) ~ Age + PS + Tobacco + HPV + strata(Cisplatin)

), data = hn_final)

# Model with log_mtv (AI-PET-GTV)

f_log_mtv <- CSC(formula = list(

  Hist(time, event) ~ Tobacco + TumorSubsite + Tstage + Nstage + log_mtv + strata(Cisplatin),

  Hist(time, event) ~ Tobacco + HPV + Tstage + Nstage + log_mtv + strata(Cisplatin),

  Hist(time, event) ~ Age + PS + Tobacco + HPV + strata(Cisplatin)

), data = hn_final)

# Save model summaries

# Save the summary of the logGTV model

output_model_logGTV <- file.path(output_analysis_folder, "output_model_logGTV.txt")

writeLines(capture.output(f_logGTV), output_model_logGTV)

cat("Summary of logGTV model saved to:", output_model_logGTV, "\n")

# Save the summary of the log_mtv (AI-PET-GTV) model

output_model_log_mtv <- file.path(output_analysis_folder, "output_model_log_mtv.txt")

writeLines(capture.output(f_log_mtv), output_model_log_mtv)

cat("Summary of log_mtv model saved to:", output_model_log_mtv, "\n")

# Step 3: Generate Score objects for each cause

performance_cause1 <- Score(

  list("logGTV model" = f_logGTV, "AI-PET-GTV model" = f_log_mtv),

  data = hn_final,

  formula = Hist(time, event) ~ 1,

  times = c(365.25, 365.25 * 3, 365.25 * 5),

  metrics = c("auc", "brier"),

  plots = c("calibration", "roc"),

  summary = "riskQuantile",

  cause = 1,  # LRF

  nullModel = FALSE

)

performance_cause2 <- Score(

  list("logGTV model" = f_logGTV, "AI-PET-GTV model" = f_log_mtv),

  data = hn_final,

  formula = Hist(time, event) ~ 1,

  times = c(365.25, 365.25 * 3, 365.25 * 5),

  metrics = c("auc", "brier"),

  plots = c("calibration", "roc"),

  summary = "riskQuantile",

  cause = 2,  # DM

  nullModel = FALSE

)

# Save performance comparison results for each cause

output_comparison_cause1 <- file.path(output_analysis_folder, "performance_comparison_cause1_results.txt")

writeLines(capture.output(performance_cause1), output_comparison_cause1)

output_comparison_cause2 <- file.path(output_analysis_folder, "performance_comparison_cause2_results.txt")

writeLines(capture.output(performance_cause2), output_comparison_cause2)

# Step 4: Generate and save ROC plots with adjusted axis labels and fonts

#roc_plot_path <- file.path(output_analysis_folder, "roc_plots_multiple_times.png")

#png(roc_plot_path, width = 1440, height = 960)

#graphics.off()  # closes any open graphics device

#roc_plot_path_tiff <- file.path(output_analysis_folder, "Fig2.tiff")

#tiff(filename = roc_plot_path_tiff, width = 1440, height = 960, units = "px", res = 600, compression = "lzw")

roc_plot_path_eps <- file.path(output_analysis_folder, "Fig2.eps")

cairo_ps(file = roc_plot_path_eps,

         width = 7, height = 4.5,

         onefile = FALSE,

         fallback_resolution = 600,

         family = "sans",

         bg = "white")

# Layout and margins

par(mfrow = c(2, 3), mar = c(4.5, 5, 3, 1))  # Slightly tighter margins

# Font sizes and line width — adjusted

par(cex.main = 0.42,

    cex.lab  = 0.36,

    cex.axis = 0.66,   # doubled tick size

    lwd      = 1.2)    # slightly thinner lines

# Plot ROC for LRF (Cause 1)

plotROC(performance_cause1, times = 365.25, plot.main = "", col = custom_colors, xlab = "", ylab = "", legend = FALSE, cex = 1, lwd=1.5)

mtext("1-year LRF prediction ROC", side = 3, line = 1, cex = 0.78)

mtext("Sensitivity", side = 2, line = 3.5, cex = 0.69)         # closer to axis

mtext("1 - Specificity", side = 1, line = 2, cex = 0.69)

plotROC(performance_cause1, times = 365.25 * 3, plot.main = "", col = custom_colors, xlab = "", ylab = "", legend = FALSE, cex = 1, lwd=1.5)

mtext("3-year LRF prediction ROC", side = 3, line = 1, cex = 0.78)

mtext("Sensitivity", side = 2, line = 3.5, cex = 0.69)

mtext("1 - Specificity", side = 1, line = 2, cex = 0.69)

plotROC(performance_cause1, times = 365.25 * 5, plot.main = "", col = custom_colors, xlab = "", ylab = "", legend = FALSE, cex = 1, lwd=1.5)

mtext("5-year LRF prediction ROC", side = 3, line = 1, cex = 0.78)

mtext("Sensitivity", side = 2, line = 3.5, cex = 0.69)

mtext("1 - Specificity", side = 1, line = 2, cex = 0.69)

# Plot ROC for DM (Cause 2)

plotROC(performance_cause2, times = 365.25, plot.main = "", col = custom_colors, xlab = "", ylab = "", legend = FALSE, cex = 1, lwd=1.5)

mtext("1-year DM prediction ROC", side = 3, line = 1, cex = 0.78)

mtext("Sensitivity", side = 2, line = 3.5, cex = 0.69)

mtext("1 - Specificity", side = 1, line = 2, cex = 0.69)

plotROC(performance_cause2, times = 365.25 * 3, plot.main = "", col = custom_colors, xlab = "", ylab = "", legend = FALSE, cex = 1, lwd=1.5)

mtext("3-year DM prediction ROC", side = 3, line = 1, cex = 0.78)

mtext("Sensitivity", side = 2, line = 3.5, cex = 0.69)

mtext("1 - Specificity", side = 1, line = 2, cex = 0.69)

plotROC(performance_cause2, times = 365.25 * 5, plot.main = "", col = custom_colors, xlab = "", ylab = "", legend = FALSE, cex = 1, lwd=1.5)

mtext("5-year DM prediction ROC", side = 3, line = 1, cex = 0.78)

mtext("Sensitivity", side = 2, line = 3.5, cex = 0.69)

mtext("1 - Specificity", side = 1, line = 2, cex = 0.69)

# Add legend with smaller font

legend("bottomright", legend = c("log(GTV) model", "log(AI-PET-GTV) model"),

       col = custom_colors, lty = 1, lwd = 1.2, cex = 0.65)

dev.off()

cat("ROC plots saved as high-resolution EPS to:", roc_plot_path_eps, "\n")

### 2non-inf-2

# Purpose: Perform non-inferiority analysis comparing AI-PET-GTV

# to manual GTV for risk stratification of LRF and DM.

# Load necessary library

library(dplyr)

# Define observed AUCs and Brier scores with their confidence intervals (Results from previous analysis in 1competing_risk_models_comparison_v2, typed in manually.)

auc_brier_data <- data.frame(

  Outcome = c("LRF", "LRF", "LRF", "DM", "DM", "DM"),  # Outcomes: LRF = loco-regional failure, DM = distant metastasis

  Time = c(365, 1096, 1826, 365, 1096, 1826),  # Timepoints in days

  # AUC values for the log(GTV) model (reference)

  auc_logGTV = c(77.0, 72.8, 70.9, 73.0, 70.8, 72.7),

  lower_logGTV = c(71.9, 67.8, 62.0, 67.8, 65.2, 62.0),

  upper_logGTV = c(82.0, 77.9, 79.8, 78.1, 76.4, 83.4),

  # AUC values for the AI-PET-GTV model (new model)

  auc_AI_PET_GTV = c(77.3, 72.9, 69.9, 77.1, 73.6, 72.2),

  lower_AI_PET_GTV = c(72.2, 67.9, 60.7, 71.8, 67.8, 58.2),

  upper_AI_PET_GTV = c(82.4, 77.9, 79.1, 82.4, 79.3, 86.2),

  # Brier scores for the log(GTV) model (reference)

  brier_logGTV = c(0.13, 0.17, 0.19, 0.07, 0.09, 0.09),

  lower_brier_logGTV = c(0.11, 0.15, 0.16, 0.05, 0.07, 0.07),

  upper_brier_logGTV = c(0.15, 0.18, 0.22, 0.08, 0.11, 0.11),

  # Brier scores for the AI-PET-GTV model (new model)

  brier_AI_PET_GTV = c(0.13, 0.17, 0.19, 0.07, 0.09, 0.10),

  lower_brier_AI_PET_GTV = c(0.11, 0.15, 0.16, 0.05, 0.07, 0.08),

  upper_brier_AI_PET_GTV = c(0.15, 0.18, 0.22, 0.08, 0.11, 0.11)

)

# Define non-inferiority margins

delta_auc <- 5.0  # Absolute 5% non-inferiority margin for AUC

delta_brier <- 0.02  # Absolute 2-point margin for Brier Score (based on previous studies)

# Compute standard error (SE) from log(GTV) confidence intervals

auc_brier_data <- auc_brier_data %>%

  mutate(

    # Standard Error (SE) for AUC computed from the log(GTV) model CI width

    SE_AUC = (upper_logGTV - lower_logGTV) / (2 * 1.96),

    # Standard Error (SE) for Brier Score computed from log(GTV) model CI width

    SE_Brier = (upper_brier_logGTV - lower_brier_logGTV) / (2 * 1.96),

    # Apply non-inferiority margin to determine the minimum acceptable threshold

    Threshold_AUC = auc_logGTV - delta_auc,

    Threshold_Brier = brier_logGTV + delta_brier,  # Higher Brier Score is worse

    # Compute Z-scores for one-tailed non-inferiority tests

    Z_Score_AUC = (auc_AI_PET_GTV - Threshold_AUC) / SE_AUC,

    Z_Score_Brier = (Threshold_Brier - brier_AI_PET_GTV) / SE_Brier,  # Higher Brier Score is worse

    # Compute one-tailed p-values

    P_Value_AUC = 1 - pnorm(Z_Score_AUC),

    P_Value_Brier = 1 - pnorm(Z_Score_Brier)

  )

# Select relevant columns for output

results <- auc_brier_data %>%

  select(Outcome, Time, Threshold_AUC, Z_Score_AUC, P_Value_AUC,

         Threshold_Brier, Z_Score_Brier, P_Value_Brier)

# Print results

print(results)

# Save results to specified output path

output_path <- "L:/LovbeskyttetMapper01/hnc-auto-cont-KHAA-DGK-data/paper_2025_analysis_biomarkers/analysis_output/non_inferiority_results_auc_brier.csv"

write.csv(results, output_path, row.names = FALSE)

### 3scatter_correlation_analysis.R

# Purpose: Generate scatter plots and compute correlation metrics

# between manual GTV and AI-PET-GTV volumes.

rm(list = ls())  # Clear the workspace

# Load required libraries

library(ggplot2)  # For plotting

library(dplyr)    # For data manipulation

library(blandr)   # For Bland-Altman analysis

library(tidyr)    # For pivot_longer

library(scales)

library(patchwork)  # For combining ggplots

# Define file paths

merged_dataset_path <- "L:\\LovbeskyttetMapper01\\hnc-auto-cont-KHAA-DGK-data\\merged_dataset.csv"

output_analysis_folder <- "L:\\LovbeskyttetMapper01\\hnc-auto-cont-KHAA-DGK-data\\paper_2025_analysis_biomarkers\\analysis_output"

# Load the merged dataset

data <- read.csv(merged_dataset_path)

data <- data[data$StudyNo != "270", ] # corrupted datapoint

# Prepare data for Figure A and B

scatter_data <- data %>%

  mutate(

    GTV = exp(logGTV),

    AI_PET_GTV = exp(log_mtv)

  )

# === Spearman Correlation Analysis ===

cor_test_result <- cor.test(

  scatter_data$GTV,

  scatter_data$AI_PET_GTV,

  method = "spearman",

  exact = FALSE

)

# Extract Spearman correlation coefficient and p-value

spearman_rho <- cor_test_result$estimate

p_value <- cor_test_result$p.value

# Print results

cat(sprintf("Spearman's rho: %.3f\n", spearman_rho))

cat(sprintf("p-value: %.3g\n", p_value))

# === Paired t-test for volume difference ===

t_test_result <- t.test(

  scatter_data$GTV,

  scatter_data$AI_PET_GTV,

  paired = TRUE

)

output_t_test_path <- file.path(output_analysis_folder, "paired_t_test_results2.txt")

writeLines(capture.output(t_test_result), output_t_test_path)

# Extract mean difference and confidence interval

mean_diff <- t_test_result$estimate

ci <- t_test_result$conf.int

p_value_ttest <- t_test_result$p.value

# Print results

cat(sprintf("Mean difference (GTV - AI-PET-GTV): %.2f cm³\n", mean_diff))

cat(sprintf("95%% CI for the difference: [%.2f, %.2f] cm³\n", ci[1], ci[2]))

cat(sprintf("p-value (paired t-test): %.3g\n", p_value_ttest))

# Find global min and max across GTV and AI-PET-GTV

axis_min <- min(c(scatter_data$GTV, scatter_data$AI_PET_GTV), na.rm = TRUE)

axis_max <- max(c(scatter_data$GTV, scatter_data$AI_PET_GTV), na.rm = TRUE)

# === Figure A: Scatter plot with identity line and equal axes ===

scatter_plot <- ggplot(scatter_data, aes(x = GTV, y = AI_PET_GTV)) +

  geom_point(aes(), shape = 21, fill = "#006BA4", color = "black", size = 2) +

  scale_fill_manual(values = c("FALSE" = "#006BA4", "TRUE" = "grey70")) +

  scale_size_manual(values = c("FALSE" = 3.5, "TRUE" = 6)) +

  scale_alpha_manual(values = c("FALSE" = 1, "TRUE" = 0.5)) +

  guides(alpha = "none", fill = "none", size = "none") +  # Hide all legends

  geom_abline(intercept = 0, slope = 1, linetype = "dashed", color = "black", linewidth = 1) +

  coord_fixed(ratio = 1, xlim = c(axis_min, axis_max), ylim = c(axis_min, axis_max)) +

  xlab("GTV volume (cm³)") +

  ylab("AI-PET-GTV volume (cm³)") +

  theme_minimal(base_size = 12) +

  theme(

    axis.text = element_text(size = 11),

    axis.title = element_text(size = 11),

    panel.background = element_rect(fill = "white", color = NA),

    plot.background = element_rect(fill = "white", color = NA),

    panel.grid = element_blank(),

    axis.line = element_line(size = 1, color = "black")

  )

# Save Figure A

output_scatter_path <- file.path(output_analysis_folder, "scatter_plot_gtv_vs_ai.eps")

ggsave(output_scatter_path, scatter_plot, device = cairo_ps, width = 7, height = 6.5, fallback_resolution = 600, bg = "white")

cat("Scatter plot saved to:", output_scatter_path, "\n")

# === Figure B: Log-Log Scatter plot with identity line and equal axes ===

scatter_plot_loglog <- ggplot(scatter_data, aes(x = GTV, y = AI_PET_GTV)) +

  geom_point(aes(), shape = 21, fill = "#006BA4", color = "black", size = 2) +

  scale_fill_manual(values = c("FALSE" = "#006BA4", "TRUE" = "grey70")) +

  scale_size_manual(values = c("FALSE" = 3.5, "TRUE" = 6)) +

  scale_alpha_manual(values = c("FALSE" = 1, "TRUE" = 0.5)) +

  guides(alpha = "none", fill = "none", size = "none") +

  geom_abline(intercept = 0, slope = 1, linetype = "dashed", color = "black", linewidth = 1) +

  scale_x_log10(

    limits = c(axis_min, axis_max),

    labels = label_number(accuracy = 1)

  ) +

  scale_y_log10(

    limits = c(axis_min, axis_max),

    labels = label_number(accuracy = 1)

  ) +

  coord_fixed(ratio = 1) +

  xlab("GTV volume (cm³, log scale)") +

  ylab("AI-PET-GTV volume (cm³, log scale)") +

  annotation_logticks(sides = "bl") +

  theme_minimal(base_size = 12) +

  theme(

    axis.text = element_text(size = 11),

    axis.title = element_text(size = 11),

    panel.background = element_rect(fill = "white", color = NA),

    plot.background = element_rect(fill = "white", color = NA),

    panel.grid = element_blank(),

    axis.line = element_line(size = 1, color = "black")

  )

# Save Figure B

output_scatter_loglog_path <- file.path(output_analysis_folder, "scatter_plot_loglog_gtv_vs_ai_v2.png")

ggsave(output_scatter_loglog_path, scatter_plot_loglog, width = 9, height = 8, dpi = 300, bg = "white")

cat("Log-log scatter plot saved to:", output_scatter_loglog_path, "\n")

# Combine plots side by side for Fig3

fig3_combined <- scatter_plot + scatter_plot_loglog +

  plot_layout(ncol = 2) & theme(plot.margin = margin(5, 5, 5, 5))  # optional margin tuning

# Save as EPS (double-column width ~17.8 cm)

output_fig3_path <- file.path(output_analysis_folder, "Fig3.eps")

ggsave(output_fig3_path,

       plot = fig3_combined,

       device = cairo_ps,

       width = 7, height = 4.5,  # inches

       fallback_resolution = 600,

       bg = "white")

cat("Combined scatter + log-log plot saved as EPS to:", output_fig3_path, "\n")

**4aalen_johansen_models_comparison_risk_based.R**

# Purpose: Generate Aalen–Johansen cumulative incidence curves

# for LRF and DM based on risk groups from competing risk models.

# Clear environment

rm(list = ls())

# Load required libraries

library(prodlim)

library(survival)

library(riskRegression)

# Define file paths

output_analysis_folder <- "L:\\LovbeskyttetMapper01\\hnc-auto-cont-KHAA-DGK-data\\paper_2025_analysis_biomarkers\\analysis_output"

# Step 1: Load the prepared dataset

hn_final <- read.csv("L:\\LovbeskyttetMapper01\\hnc-auto-cont-KHAA-DGK-data\\merged_dataset.csv")

hn_final <- hn_final[hn_final$StudyNo != "270", ]

# Ensure the dataset contains required variables

if (!all(c("time", "event", "logGTV", "log_mtv") %in% colnames(hn_final))) {

  stop("The dataset does not contain all required variables.")

}

# change time to years

hn_final$time <- hn_final$time / 365.25

hn_final$Tobacco <- relevel(factor(hn_final$Tobacco), ref = "Never/previous smoker")

hn_final$TumorSubsite <- relevel(factor(hn_final$TumorSubsite), ref = "Oropharynx, p16 positive")

hn_final$Nstage <- relevel(factor(hn_final$Nstage), ref = "N0")

hn_final$HPV <- relevel(factor(hn_final$HPV), ref = "p16+ oropharynx")

hn_final$Cisplatin <- relevel(factor(hn_final$Cisplatin), ref = "No cisplatin")

# Ensure the dataset contains required variables

required_vars <- c("time", "event", "logGTV", "log_mtv")

if (!all(required_vars %in% colnames(hn_final))) {

  stop("The dataset does not contain all required variables.")

}

# Step 2: Define Cox models

# Model with logGTV (manual GTV)

f_logGTV <- CSC(formula = list(

  Hist(time, event) ~ Tobacco + TumorSubsite + Tstage + Nstage + logGTV + strata(Cisplatin),

  Hist(time, event) ~ Tobacco + HPV + Tstage + Nstage + logGTV + strata(Cisplatin),

  Hist(time, event) ~ Age + PS + Tobacco + HPV + strata(Cisplatin)

), data = hn_final)

# Model with log_mtv (AI-PET-GTV)

f_log_mtv <- CSC(formula = list(

  Hist(time, event) ~ Tobacco + TumorSubsite + Tstage + Nstage + log_mtv + strata(Cisplatin),

  Hist(time, event) ~ Tobacco + HPV + Tstage + Nstage + log_mtv + strata(Cisplatin),

  Hist(time, event) ~ Age + PS + Tobacco + HPV + strata(Cisplatin)

), data = hn_final)

# Step 3: Compute predicted risks using the competing risk models for LRF and DM

# Predict risks for LRF (Cause 1)

hn_final$logGTV_predicted_risk_LRF <- predictRisk(f_logGTV, cause = 1, times=3,se=TRUE, newdata = hn_final)

hn_final$log_mtv_predicted_risk_LRF <- predictRisk(f_log_mtv, cause = 1, times=3,se=TRUE, newdata = hn_final)

# Predict risks for DM (Cause 2)

hn_final$logGTV_predicted_risk_DM <- predictRisk(f_logGTV, cause = 2, times=3,se=TRUE, newdata = hn_final)

hn_final$log_mtv_predicted_risk_DM <- predictRisk(f_log_mtv, cause = 2, times=3,se=TRUE, newdata = hn_final)

# Step 6: Compare stratification of top 25% high-risk patients based on predicted risk

# Compute the 4th median cutoff for predicted risks

median_logGTV_LRF <- quantile(hn_final$logGTV_predicted_risk_LRF, probs = 0.5, na.rm = TRUE)

median_log_mtv_LRF <- quantile(hn_final$log_mtv_predicted_risk_LRF, probs = 0.5, na.rm = TRUE)

median_logGTV_DM <- quantile(hn_final$logGTV_predicted_risk_DM, probs = 0.5, na.rm = TRUE)

median_log_mtv_DM <- quantile(hn_final$log_mtv_predicted_risk_DM, probs = 0.5, na.rm = TRUE)

# Categorize patients into high and low risk based on the cutoffs

hn_final$logGTV_LRF_category <- ifelse(hn_final$logGTV_predicted_risk_LRF > median_logGTV_LRF, "high", "low")

hn_final$log_mtv_LRF_category <- ifelse(hn_final$log_mtv_predicted_risk_LRF > median_log_mtv_LRF, "high", "low")

hn_final$logGTV_DM_category <- ifelse(hn_final$logGTV_predicted_risk_DM > median_logGTV_DM, "high", "low")

hn_final$log_mtv_DM_category <- ifelse(hn_final$log_mtv_predicted_risk_DM > median_log_mtv_DM, "high", "low")

# Ensure categories are treated as factors with "low" first

hn_final$logGTV_LRF_category <- factor(hn_final$logGTV_LRF_category, levels = c("low", "high"))

hn_final$log_mtv_LRF_category <- factor(hn_final$log_mtv_LRF_category, levels = c("low", "high"))

hn_final$logGTV_DM_category <- factor(hn_final$logGTV_DM_category, levels = c("low", "high"))

hn_final$log_mtv_DM_category <- factor(hn_final$log_mtv_DM_category, levels = c("low", "high"))

# Add nonparametric (Aalen–Johansen) curves in green - for LRF

aj_fit_GTV_LRF <- prodlim(Hist(time, event) ~ logGTV_LRF_category, data = hn_final, type = "risk")

aj_fit_mtv_LRF <- prodlim(Hist(time, event) ~ log_mtv_LRF_category, data = hn_final, type = "risk")

# add nonparametric (Aalen–Johansen) curves in green - for DM

aj_fit_GTV_DM <- prodlim(Hist(time, event) ~ logGTV_DM_category, data = hn_final, type = "risk")

aj_fit_mtv_DM <- prodlim(Hist(time, event) ~ log_mtv_DM_category, data = hn_final, type = "risk")

# save fit results to a text file.

output_aj_fit_path <- file.path(output_analysis_folder, "aalen_johansen_log_mtv_results.txt")

options(width = 200)

aj_fit_output <- capture.output({

    cat("===== Aalen-Johansen Results: log_mtv_LRF_category =====\n\n")

    print(summary(aj_fit_mtv_LRF),times=3)

    cat("\n\n===== Aalen-Johansen Results: logGTV_LRF_category =====\n\n")

    print(summary(aj_fit_GTV_LRF),times=3)

    cat("\n\n===== Aalen-Johansen Results: log_mtv_DM_category =====\n\n")

    print(summary(aj_fit_mtv_DM),times=3)

    cat("\n\n===== Aalen-Johansen Results: logGTV_DM_category =====\n\n")

    print(summary(aj_fit_GTV_DM),times=3)

})

writeLines(aj_fit_output, output_aj_fit_path)

cat("Aalen-Johansen results for both log_mtv_category and logGTV_category saved to:", output_aj_fit_path, "\n")

# Step 4: Plot Adjusted CIFs and add Aalen–Johansen (nonparametric) curves

# Define EPS output path

aalen_johansen_plot_eps_path <- file.path(output_analysis_folder, "Fig4.eps")

# Open EPS device: wider than tall

cairo_ps(file = aalen_johansen_plot_eps_path,

         width = 7.5, height = 3.5,

         onefile = FALSE,

         fallback_resolution = 600,

         family = "sans")

# Set tight margins, small labels, and axis spacing

par(mfrow = c(1, 2),                     # 1 row, 2 columns

    mar = c(4.2, 4.6, 2.4, 1.5),         # bottom, left, top, right

    mgp = c(2.2, 0.6, 0),                # axis title, label, line

    cex.main = 0.9,

    cex.lab = 0.8,

    cex.axis = 0.6,                    # 30% smaller than your previous 0.7

    lwd = 1.4)

# Define manual tick marks

y_ticks <- seq(0, 0.45, by = 0.1)

x_ticks <- pretty(hn_final$time, n = 5)  # adjust if needed

# Panel A: LRF

plot(aj_fit_GTV_LRF, cause = 1, col = "#0858B3", type = "risk",

     lwd = 1.5, lty = c(3,1), confint = FALSE, ylim = c(0, 0.45),

     ylab = "", xlab = "", legend = FALSE, axes = FALSE, atrisk = FALSE,

     background = TRUE,

     background.horizontal = FALSE)

plot(aj_fit_mtv_LRF, cause = 1, col = "#2E8B57", type = "risk",

     lwd = 1.5, lty = c(3,1), confint = FALSE, ylim = c(0, 0.45), add = TRUE)

abline(h = c(0.1, 0.2, 0.3, 0.4), col = "gray80", lwd = 1)

axis(1); axis(2)

legend("bottomright",

       legend = c("High risk (GTV model)", "Low risk (GTV model)",

                  "High risk (AI-PET-GTV model)", "Low risk (AI-PET-GTV model)"),

       col = c("#0858B3", "#0858B3", "#2E8B57", "#2E8B57"),

       lwd = 1.5, lty = c(1, 3, 1, 3), cex = 0.5, bty = "o", bg= "white")

mtext("Loco-regional Failure (LRF)", side = 3, line = 0.8, cex = 0.9)

mtext("Time (years)", side = 1, line = 2.4, cex = 0.7)

mtext("Absolute Risk", side = 2, line = 2.8, cex = 0.7)

# Panel B: DM

plot(aj_fit_GTV_DM, cause = 2, group = "high", col = "#0858B3", type = "risk",

     lwd = 1.5, lty = c(3,1), confint = FALSE, ylim = c(0, 0.20),

     ylab = "", xlab = "", legend = FALSE, axes = FALSE, atrisk = FALSE)

plot(aj_fit_mtv_DM, cause = 2, col = "#2E8B57", type = "risk",

     lwd = 1.5, lty = c(3,1), confint = FALSE, add = TRUE)

axis(1); axis(2)

mtext("Distant Metastases (DM)", side = 3, line = 0.8, cex = 0.9)

mtext("Time (years)", side = 1, line = 2.4, cex = 0.7)

mtext("Absolute Risk", side = 2, line = 2.8, cex = 0.7)

# Close device

dev.off()

cat("Final Fig 4 EPS saved to:", aalen_johansen_plot_eps_path, "\n")

# Define exact 3-year time point

time_3_years <- 3

# Use summary.prodlim to extract cumulative incidence at 3 years

ci_3y_aj_GTV     <- summary(aj_fit_GTV_LRF, times = time_3_years, cause = 1)  # GTV for LRF

ci_3y_aj_mtv     <- summary(aj_fit_mtv_LRF, times = time_3_years, cause = 1)  # AI-PET-GTV for LRF

ci_3y_aj_GTV_DM  <- summary(aj_fit_GTV_DM,  times = time_3_years, cause = 2)  # GTV for DM

ci_3y_aj_mtv_DM  <- summary(aj_fit_mtv_DM,  times = time_3_years, cause = 2)  # AI-PET-GTV for DM

# Save extracted results

output_3y_path <- file.path(output_analysis_folder, "aalen_johansen_3year_results.txt")

options(width = 200)

ci_3y_output <- capture.output({

  cat("===== 3-Year Cumulative Incidence Estimates (Aalen-Johansen) =====\n\n")

  cat("**Locoregional Failure (LRF) - AI-PET-GTV**\n")

  print(ci_3y_aj_mtv)

  cat("\n**Locoregional Failure (LRF) - GTV**\n")

  print(ci_3y_aj_GTV)

  cat("\n**Distant Metastases (DM) - AI-PET-GTV**\n")

  print(ci_3y_aj_mtv_DM)

  cat("\n**Distant Metastases (DM) - GTV**\n")

  print(ci_3y_aj_GTV_DM)

})

writeLines(ci_3y_output, output_3y_path)

cat("3-Year Aalen-Johansen estimates saved to:", output_3y_path, "\n")

### 5risk_volume_relationship.R

# Purpose: Assess the relationship between predicted risk and tumour volume

# by comparing GTV and AI-PET-GTV distributions in high vs. low risk groups.

rm(list = ls())

library(prodlim)

library(survival)

library(gridExtra)

library(scales)

output_analysis_folder <- "L:\\LovbeskyttetMapper01\\hnc-auto-cont-KHAA-DGK-data\\paper_2025_analysis_biomarkers\\analysis_output"

# Step 1: Load the prepared dataset

hn_final <- read.csv("L:\\LovbeskyttetMapper01\\hnc-auto-cont-KHAA-DGK-data\\merged_dataset.csv")

# due to data corruption we have to remove the study 270

hn_final <- hn_final[hn_final$StudyNo != "270", ]

hn_final$Tobacco <- relevel(factor(hn_final$Tobacco), ref = "Never/previous smoker")

hn_final$TumorSubsite <- relevel(factor(hn_final$TumorSubsite), ref = "Oropharynx, p16 positive")

hn_final$Nstage <- relevel(factor(hn_final$Nstage), ref = "N0")

hn_final$HPV <- relevel(factor(hn_final$HPV), ref = "p16+ oropharynx")

hn_final$Cisplatin <- relevel(factor(hn_final$Cisplatin), ref = "No cisplatin")

# Ensure the dataset contains required variables

required_vars <- c("time", "event", "logGTV", "log_mtv")

if (!all(required_vars %in% colnames(hn_final))) {

  stop("The dataset does not contain all required variables.")

}

# Step 2: Define Cox models

# Model with logGTV (manual GTV)

f_logGTV <- CSC(formula = list(

  Hist(time, event) ~ Tobacco + TumorSubsite + Tstage + Nstage + logGTV + strata(Cisplatin),

  Hist(time, event) ~ Tobacco + HPV + Tstage + Nstage + logGTV + strata(Cisplatin),

  Hist(time, event) ~ Age + PS + Tobacco + HPV + strata(Cisplatin)

), data = hn_final)

# Model with log_mtv (AI-PET-GTV)

f_log_mtv <- CSC(formula = list(

  Hist(time, event) ~ Tobacco + TumorSubsite + Tstage + Nstage + log_mtv + strata(Cisplatin),

  Hist(time, event) ~ Tobacco + HPV + Tstage + Nstage + log_mtv + strata(Cisplatin),

  Hist(time, event) ~ Age + PS + Tobacco + HPV + strata(Cisplatin)

), data = hn_final)

# Step 3: Compute predicted risks using the competing risk models for LRF and DM

# Predict risks for LRF (Cause 1)

hn_final$logGTV_predicted_risk_LRF <- predictRisk(f_logGTV, cause = 1, times=365.25*3,se=TRUE, newdata = hn_final)

hn_final$log_mtv_predicted_risk_LRF <- predictRisk(f_log_mtv, cause = 1, times=365.25*3,se=TRUE, newdata = hn_final)

# Predict risks for DM (Cause 2)

hn_final$logGTV_predicted_risk_DM <- predictRisk(f_logGTV, cause = 2, times=365.25*3,se=TRUE, newdata = hn_final)

hn_final$log_mtv_predicted_risk_DM <- predictRisk(f_log_mtv, cause = 2, times=365.25*3,se=TRUE, newdata = hn_final)

# Step 6: Compare stratification of top 25% high-risk patients based on predicted risk

# Compute the 4th median cutoff for predicted risks

median_logGTV_LRF <- quantile(hn_final$logGTV_predicted_risk_LRF, probs = 0.5, na.rm = TRUE)

median_log_mtv_LRF <- quantile(hn_final$log_mtv_predicted_risk_LRF, probs = 0.5, na.rm = TRUE)

median_logGTV_DM <- quantile(hn_final$logGTV_predicted_risk_DM, probs = 0.5, na.rm = TRUE)

median_log_mtv_DM <- quantile(hn_final$log_mtv_predicted_risk_DM, probs = 0.5, na.rm = TRUE)

# Categorize patients into high and low risk based on the cutoffs

hn_final$logGTV_LRF_category <- ifelse(hn_final$logGTV_predicted_risk_LRF > median_logGTV_LRF, "high", "low")

hn_final$log_mtv_LRF_category <- ifelse(hn_final$log_mtv_predicted_risk_LRF > median_log_mtv_LRF, "high", "low")

hn_final$logGTV_DM_category <- ifelse(hn_final$logGTV_predicted_risk_DM > median_logGTV_DM, "high", "low")

hn_final$log_mtv_DM_category <- ifelse(hn_final$log_mtv_predicted_risk_DM > median_log_mtv_DM, "high", "low")

# Define colors

color_high <- "#B30838"  # Red for high-risk groups

color_low <- "#0858B3"   # Blue for low-risk groups

# Ensure categories are treated as factors with "low" first

hn_final$logGTV_LRF_category <- factor(hn_final$logGTV_LRF_category, levels = c("low", "high"))

hn_final$log_mtv_LRF_category <- factor(hn_final$log_mtv_LRF_category, levels = c("low", "high"))

hn_final$logGTV_DM_category <- factor(hn_final$logGTV_DM_category, levels = c("low", "high"))

hn_final$log_mtv_DM_category <- factor(hn_final$log_mtv_DM_category, levels = c("low", "high"))

# Convert log-transformed values back to actual tumor volumes

hn_final$GTV_volume <- exp(hn_final$logGTV)

hn_final$AI_PET_GTV_volume <- exp(hn_final$log_mtv)

# Determine a common y-axis range for all plots (using actual volumes)

y_limits <- range(c(hn_final$GTV_volume, hn_final$AI_PET_GTV_volume), na.rm = TRUE)

# ---- BOXPLOTS ----

# LRF-based risk stratification

p1 <- ggplot(hn_final, aes(x = logGTV_LRF_category, y = GTV_volume, fill = logGTV_LRF_category)) +

  geom_boxplot(size = 0.8, outlier.size = 1.5, notch = TRUE) +

  scale_fill_manual(values = c(color_low, color_high)) +

  scale_y_log10(limits = y_limits, labels = scales::comma) +

  labs(title = "LRF - GTV-Based Model", x = "Risk Group", y = "GTV Volume (cm³) (Log Scale)") +

  theme_minimal(base_size = 12) +

  theme(legend.position = "none", panel.grid = element_blank(), axis.line = element_line(size = 1, color = "black"),

  plot.title = element_text(size = 12),

  axis.title = element_text(size = 11),

  axis.text = element_text(size = 10))

p2 <- ggplot(hn_final, aes(x = log_mtv_LRF_category, y = AI_PET_GTV_volume, fill = log_mtv_LRF_category)) +

  geom_boxplot(size = 0.8, outlier.size = 1.5, notch = TRUE) +

  scale_fill_manual(values = c(color_low, color_high)) +

  scale_y_log10(limits = y_limits, labels = scales::comma) +

  labs(title = "LRF - AI-PET-GTV Model", x = "Risk Group", y = "AI-PET-GTV Volume (cm³) (Log Scale)") +

  theme_minimal(base_size = 12) +

  theme(legend.position = "none", panel.grid = element_blank(), axis.line = element_line(size = 1, color = "black"),

  plot.title = element_text(size = 12),

  axis.title = element_text(size = 11),

  axis.text = element_text(size = 10))

# DM-based risk stratification

p3 <- ggplot(hn_final, aes(x = logGTV_DM_category, y = GTV_volume, fill = logGTV_DM_category)) +

  geom_boxplot(size = 0.8, outlier.size = 1.5, notch = TRUE) +

  scale_fill_manual(values = c(color_low, color_high)) +

  scale_y_log10(limits = y_limits, labels = scales::comma) +

  labs(title = "DM - GTV-Based Model", x = "Risk Group", y = "GTV Volume (cm³) (Log Scale)") +

  theme_minimal(base_size = 12) +

  theme(legend.position = "none", panel.grid = element_blank(), axis.line = element_line(size = 1, color = "black"),

  plot.title = element_text(size = 12),

  axis.title = element_text(size = 11),

  axis.text = element_text(size = 10))

p4 <- ggplot(hn_final, aes(x = log_mtv_DM_category, y = AI_PET_GTV_volume, fill = log_mtv_DM_category)) +

  geom_boxplot(size = 0.8, outlier.size = 1.5, notch = TRUE) +

  scale_fill_manual(values = c(color_low, color_high)) +

  scale_y_log10(limits = y_limits, labels = scales::comma) +

  labs(title = "DM - AI-PET-GTV Model", x = "Risk Group", y = "AI-PET-GTV Volume (cm³) (Log Scale)") +

  theme_minimal(base_size = 12) +

  theme(legend.position = "none", panel.grid = element_blank(), axis.line = element_line(size = 1, color = "black"),

  plot.title = element_text(size = 12),

  axis.title = element_text(size = 11),

  axis.text = element_text(size = 10))

# Define EPS output path

eps_path <- file.path(output_analysis_folder, "Fig5.eps")

# Save as EPS using cairo_ps

cairo_ps(file = eps_path, width = 7, height = 7, onefile = FALSE, fallback_resolution = 600)

grid.arrange(p1, p2, p3, p4, ncol = 2, nrow = 2)

dev.off()

cat("Figure 5 EPS file saved to:", eps_path, "\n")

median(hn_final$GTV_volume[hn_final$logGTV_LRF_category == "high"])

median(hn_final$AI_PET_GTV_volume[hn_final$log_mtv_LRF_category == "high"])

median(hn_final$GTV_volume[hn_final$logGTV_LRF_category == "low"])

median(hn_final$AI_PET_GTV_volume[hn_final$log_mtv_LRF_category == "low"])

median(hn_final$GTV_volume[hn_final$logGTV_DM_category == "high"])

median(hn_final$GTV_volume[hn_final$logGTV_DM_category == "low"])

median(hn_final$AI_PET_GTV_volume[hn_final$log_mtv_DM_category == "high"])

median(hn_final$AI_PET_GTV_volume[hn_final$log_mtv_DM_category == "low"])

# Helper function

print_median_iqr <- function(values, label) {

  med <- median(values, na.rm = TRUE)

  iqr_vals <- quantile(values, probs = c(0.25, 0.75), na.rm = TRUE)

  cat(sprintf(

    "%s:\n  Median = %.1f cm³\n  IQR = [%.1f – %.1f] cm³\n\n",

    label, med, iqr_vals[1], iqr_vals[2]

  ))

}

# === LOCORREGIONAL FAILURE (LRF) ===

print_median_iqr(hn_final$GTV_volume[hn_final$logGTV_LRF_category == "high"],   "LRF - GTV (High Risk)")

print_median_iqr(hn_final$AI_PET_GTV_volume[hn_final$log_mtv_LRF_category == "high"], "LRF - AI-PET-GTV (High Risk)")

print_median_iqr(hn_final$GTV_volume[hn_final$logGTV_LRF_category == "low"],    "LRF - GTV (Low Risk)")

print_median_iqr(hn_final$AI_PET_GTV_volume[hn_final$log_mtv_LRF_category == "low"],  "LRF - AI-PET-GTV (Low Risk)")

# === DISTANT METASTASIS (DM) ===

print_median_iqr(hn_final$GTV_volume[hn_final$logGTV_DM_category == "high"],    "DM - GTV (High Risk)")

print_median_iqr(hn_final$AI_PET_GTV_volume[hn_final$log_mtv_DM_category == "high"],  "DM - AI-PET-GTV (High Risk)")

print_median_iqr(hn_final$GTV_volume[hn_final$logGTV_DM_category == "low"],     "DM - GTV (Low Risk)")

print_median_iqr(hn_final$AI_PET_GTV_volume[hn_final$log_mtv_DM_category == "low"],   "DM - AI-PET-GTV (Low Risk)")

# ---- T-TESTS ----

# (not reported in paper, since this is more of a characterization of the data, but its also clear from the figures that the differenes are significant)

# LRF Risk Groups

t_test_LRF_GTV <- t.test(GTV_volume ~ logGTV_LRF_category, data = hn_final)

t_test_LRF_mtv <- t.test(AI_PET_GTV_volume ~ log_mtv_LRF_category, data = hn_final)

# DM Risk Groups

t_test_DM_GTV <- t.test(GTV_volume ~ logGTV_DM_category, data = hn_final)

t_test_DM_mtv <- t.test(AI_PET_GTV_volume ~ log_mtv_DM_category, data = hn_final)

# Save t-test results

t_test_results_path <- file.path(output_analysis_folder, "t_test_results.txt")

sink(t_test_results_path)

cat("T-Test Results for Tumor Volume Differences Between High and Low-Risk Groups\n\n")

cat("LRF - GTV Model:\n")

print(t_test_LRF_GTV)

cat("\nLRF - AI-PET-GTV Model:\n")

print(t_test_LRF_mtv)

cat("\nDM - GTV Model:\n")

print(t_test_DM_GTV)

cat("\nDM - AI-PET-GTV Model:\n")

print(t_test_DM_mtv)

sink()

cat("T-test results saved to:", t_test_results_path, "\n")
